# Automated classification of hip fractures using deep convolutional neural networks with orthopedic surgeon-level accuracy: ensemble decision-making with antero-posterior and lateral radiographs

**DOI:** 10.1080/17453674.2020.1803664

**Published:** 2020-08-12

**Authors:** Yutoku Yamada, Satoshi Maki, Shunji Kishida, Haruki Nagai, Junnosuke Arima, Nanako Yamakawa, Yasushi Iijima, Yuki Shiko, Yohei Kawasaki, Toshiaki Kotani, Yasuhiro Shiga, Kazuhide Inage, Sumihisa Orita, Yawara Eguchi, Hiroshi Takahashi, Takeshi Yamashita, Shohei Minami, Seiji Ohtori

**Affiliations:** a Department of Orthopaedic Surgery, Graduate School of Medicine, Chiba University, Japan; b Department of Orthopaedic Surgery, Seirei Sakura Citizen Hospital; c Department of Orthopaedic Surgery, Oyumino Central Hospital; d Biostatistics Section, Clinical Research Center, Chiba University Hospital; e Center for Frontier Medical Engineering, Chiba University; f Department of Orthopaedic Surgery, Toho University Sakura Medical Center, Japan

## Abstract

Background and purpose — Deep-learning approaches based on convolutional neural networks (CNNs) are gaining interest in the medical imaging field. We evaluated the diagnostic performance of a CNN to discriminate femoral neck fractures, trochanteric fractures, and non-fracture using antero-posterior (AP) and lateral hip radiographs.

Patients and methods — 1,703 plain hip AP radiographs and 1,220 plain hip lateral radiographs were included in the total dataset. 150 images each of the AP and lateral views were separated out and the remainder of the dataset was used for training. The CNN made the diagnosis based on: (1) AP radiographs alone, (2) lateral radiographs alone, or (3) both AP and lateral radiographs combined. The diagnostic performance of the CNN was measured by the accuracy, recall, precision, and F1 score. We further compared the CNN’s performance with that of orthopedic surgeons.

Results — The average accuracy, recall, precision, and F1 score of the CNN based on both anteroposterior and lateral radiographs were 0.98, 0.98, 0.98, and 0.98, respectively. The accuracy of the CNN was comparable to, or statistically significantly better than, that of the orthopedic surgeons regardless of radiographic view used. In the CNN model, the accuracy of the diagnosis based on both views was significantly better than the lateral view alone and tended to be better than the AP view alone.

Interpretation — The CNN exhibited comparable or superior performance to that of orthopedic surgeons to discriminate femoral neck fractures, trochanteric fractures, and non-fracture using both AP and lateral hip radiographs.

Although conventional radiography is the mainstay for diagnosing fractures, the sensitivity of radiographs to detect hip fracture is not ideal. Previous reports have indicated that the rate of initial misdiagnosis varies between 7% and 14% (Chellam 2016), and delayed diagnosis and treatment may lead to malunion, osteonecrosis, and arthritis, resulting in a poor prognosis (Parker 1992).

Deep learning is a branch of machine learning that has recently yielded breakthroughs in computer vision tasks. A deep-learning approach based on convolutional neural networks (CNNs) is gaining interest across a variety of domains including medical imaging. CNNs are designed to automatically and adaptively learn features from data through backpropagation by using multiple building blocks, such as convolution layers, pooling layers, and fully connected layers (Greenspan et al. 2016). Owing to large datasets and increased computing power, CNNs have rapidly become a cutting-edge method for enhancing performance in medical image analysis. Recently, an increasing number of clinical applications have been reported in radiology for detection, classification, and segmentation tasks. However, studies using CNNs in the field of orthopedic surgery and traumatology are limited and the field is immature. So far, there are radiographic studies using CNNs for hip fractures (Adams et al. 2019, Badgeley et al. 2019, Cheng et al. 2019, Urakawa et al. 2019), distal radius fractures (Kim and MacKinnon 2018, Gan et al. 2019, Yahalomi et al. 2019, Blüthgen et al. 2020), proximal humeral fractures (Chung et al. 2018), ankle fractures (Kitamura et al. 2019) and hand, wrist, and ankle fractures (Olczak et al. 2017).

This study evaluates the diagnostic performance of a CNN for detecting and classifying hip fractures using plain antero-posterior (AP) and lateral hip radiographs. We compared the diagnostic performance with that of orthopedic surgeons.

## Patients and methods

### Patients

We retrospectively reviewed the medical records of all consecutive patients with hip fractures who were admitted to the Seirei Sakura Citizen Hospital between April 2015 and January 2020 and the Oyumino Central Hospital between March 2014 and January 2019. Diagnosis of the fracture type was made mainly using radiographs and computed tomography by at least 2 board-certified orthopedic surgeons. Particularly for the cases where the fracture pattern was not clear, we also used MRI to make the diagnosis. Basal neck fractures were classified as trochanteric fractures because they are recommended to be treated as an extra-capsular fracture using a sliding hip screw (Mallick and Parker 2004). Other fractures, including femoral head and subtrochanteric fractures, were not included in the study. There were 569 patients with femoral neck fracture and 466 patients with trochanteric fracture. The radiographs of non-fractured hips in the AP view were obtained from the AP radiographs of the hip contralateral to the fractured hip. The radiographs of non-fractured hips in the lateral view were obtained from patients with suspected hip fractures that were diagnosed as sprains or bruises of the hip joint.

### Radiographic dataset

Poor-quality images, such as those with poor image contrast (16 AP images and 24 lateral images), anatomical side markers in the region (34 AP images and 5 lateral images), foreign body interference (8 AP images and 2 lateral images), and metal implants (105 AP images) were excluded. Moreover, 196 AP radiographs of non-fractured hips were chosen randomly and excluded to avoid the problem of imbalanced classes. The dataset used in this study included 1,703 plain hip AP radiographs (556 femoral neck fracture cases, 441 trochanteric fracture cases, and 706 normal hips) from 1,047 patients (801 women, 246 men; 567 from Seirei Sakura Citizen Hospital, 480 from Oyumino Central Hospital) and 1,220 plain hip lateral radiographs (555 femoral neck fracture cases, 431 trochanteric fracture cases, and 234 normal hips) from 1,220 patients (911 women, 309 men; 818 from Seirei Sakura Citizen Hospital, 402 from Oyumino Central Hospital). We used only 1 image per patient to decrease the overfitting of the CNN except for the cases of non-fracture AP radiographs. We reserved 50 of the AP and lateral radiographs from each set of femoral neck fractures, trochanteric fractures, and non-fractures for the validation dataset (i.e., 150 images each for AP and lateral views) and used the remainder of the dataset for training. The validation dataset was taken from the latest patient admitted to the Seirei Sakura Citizen Hospital during the study period.

### Image preprocessing for deep learning

**Figure 1.**
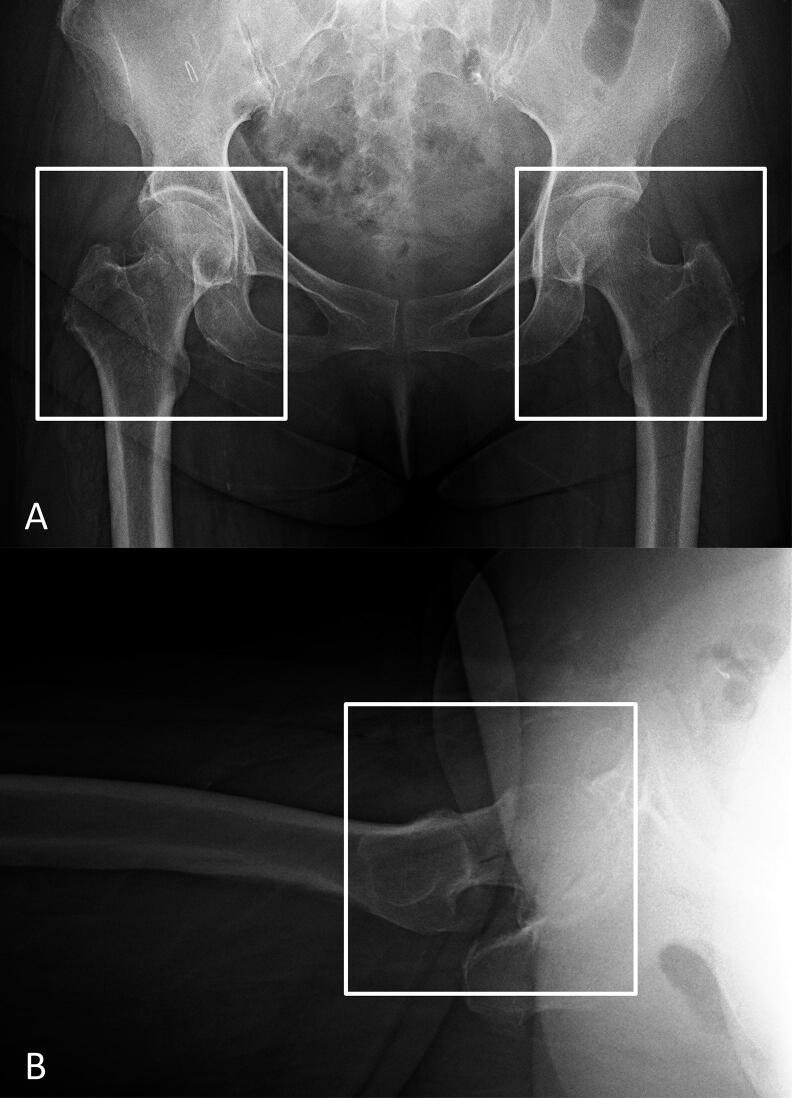
Image preprocessing for the convolutional neural network model training and validation. We cropped images to a minimum region containing the femoral head and the greater and lesser trochanters in both the AP (A) and lateral (B) hip radiographs. On the AP radiographs, the fractured hip (left white box) was cropped and the side contralateral from the fractured hip (right white box) was cropped as the non-fractured hip. AP = anteroposterior.

Plain antero-posterior (AP) and lateral hip radiographs from digital imaging and communications in medicine (DICOM) files were exported in jpeg format from the picture archiving and communication systems (PACS) in our hospital. An orthopedic surgeon (YY, 3 years of experience) performed the image preprocessing using Paint 3D (Microsoft Corp, Redmond, WA, USA) by cropping the minimum region containing the femoral head and greater and lesser trochanters on both exported AP and lateral hip radiographs to generate an image for the CNN training (Figure 1).

### Model construction and training of the CNN

Python programming language, version 3.6.7 (https://www.python.org) and Keras, version 2.2.4 with Tensorflow, version 1.14.0 (https://www.tensorflow.org) at the backend were used to construct the CNN architecture. We used the Xception architectural model, which had been previously trained using images with ImageNet. The Xception architecture has 36 convolutional layers forming the feature extraction base of the network. The 36 convolutional layers are structured into 14 modules, all of which have linear residual connections around them, except for the first and last modules. In short, the Xception architecture is a linear stack of depth-wise separable convolution layers with residual connections, which makes the architecture easy to define and modify (Chollet 2016). The input images were scaled down to 299 × 299 pixels. We then fine-tuned the model with the dataset of radiographs of femoral neck and trochanteric fractures, as well as non-fractures. Weights in the first 108 layers were frozen and weights in the other layers were retrained with our data. The network was trained for 100 epochs with a learning rate of 0.1, which was reduced if no improvement was seen. Convergence of the model training was monitored using cross-entropy loss. All images were randomly augmented using ImageDataGenerator (https://keras.io/preprocessing/image/) by a rotation angle range of 20°, width shift range of 0.2, height shift range of 0.2, brightness range of 0.3–1.0, and a horizontal flip of 50%. In addition, the models were separately constructed for AP and lateral radiographic views. The CNN was trained and validated using a computer with a GeForce RTX 2060 graphics processing unit (NVIDIA, Santa Clara, CA, USA), a Core i7-9750 central processing unit (Intel, Santa Clara, CA, USA), and 16 GB of random-access memory.

### Performance evaluation

The performance of the CNN model for differentiating femoral neck fractures from trochanteric fractures and non-fracture was evaluated using the validation dataset, which was not included in the training dataset. The performance of the CNN was evaluated in 3 ways and a diagnosis was made for each: (1) AP hip radiographs alone; (2) lateral hip radiographs alone; (3) both AP and lateral hip radiographs. The probabilities for femoral neck fractures, trochanteric fractures, and fractures were determined for each view. The final decision was made based on the highest probability score between the 3 diagnoses. When diagnosing the fracture based on both views, the probability of the diagnosis on the AP and lateral view was averaged and final diagnosis was made based on the highest probability score. This enabled a comprehensive decision based on both the AP and lateral views and not based on a single view, which is similar to the way clinicians make a diagnosis from radiographs.

### Image assessment by orthopedic surgeons

2 board-certified orthopedic surgeons (SK and SM with 22 and 14 years of experience, respectively) and 2 resident orthopedic surgeons (NY and JA with 4 and 3 years of experience, respectively) reviewed the AP and lateral hip radiographs in jpeg format, which had an identical area to those used during the training of the CNN but with the same resolution as the original DICOM image. This was intended to provide fair competition between the CNN and the clinicians, although this situation differs from the clinical setting. They reviewed the hip radiographs in the same 3 ways as the CNN and made a diagnosis for each method: (1) AP hip radiographs alone; (2) lateral hip radiographs; alone (3) both the AP and lateral hip radiographs together. The readers were blinded to clinical information such as the age of the patient and the mechanism of injury.

### Statistical and data analyses

All statistical analyses were conducted using SAS (version 9.4 for Windows) and JMP (version 12.0.1; SAS Institute Inc., Cary, NC, USA). Continuous variables were evaluated using an analysis of variance (ANOVA), and categorical variables were evaluated using a chi-square test. A threshold of p < 0.05 was considered significant in two-sided tests of statistical inference. Inter-rater reliability for fractures was calculated using Cohen’s kappa between the orthopedic surgeons. We calculated the true positive (TP), true negative (TN), false positive (TP), and false negative (FN) rates based on the predictions of the CNN and orthopedic surgeons. To evaluate the performance, we calculated the average values of accuracy, recall, precision, and F1 score. The accuracy, recall, precision, and F1 score were calculated by the following numerical formula: Accuracy = TP + TN/TP + FP + FN + TN; Recall = TP/TP + FN; Precision = TP/TP + FP; F1score = 2 × Recall × Precision/Recall + Precision. The accuracy of the diagnostic performance of the CNN and the orthopedic surgeons was compared using a McNemar test. The accuracy of diagnostic performance difference between the radiographic views (i.e., AP, lateral, and 2 views) was also compared using a McNemar test.

### Ethics, funding, and potential conflicts of interest

The study was approved by the Institutional Review Board of the 3 institutions involved and the requirement for consent was waived because of the retrospective analysis (IRB no. 3329). This work was supported by a research grant funded by the Japanese Orthopaedic Association and Inohana-Shougakukai Grants-in-Aid. The authors report no conflict of interest concerning the materials or methods used in this study or the findings specified in this article.

## Results

### Patient characteristics

The non-fracture group consisted of patients who had normal lateral hip radiographs. The number of non-fracture images was relatively small because obtaining lateral radiographs of a non-fractured hip is difficult (Table 1). The non-fracture group was younger than the patients with femoral neck fractures and trochanteric fractures. There were 6 cases of femoral neck fracture and 5 of trochanteric fracture that could not be diagnosed by radiographs and CT and were subsequently diagnosed by MRI.

**Table 1.** Baseline patient characteristics

Factor	Femoral neck fracture n = 569	Trochanteric fracture n = 466	Non-fracture n = 234
Age, mean (SD)	81.3 (11.4)	85.2 (10.0)	68.8 (16.2)
Sex (M/F), n	136/433	105/361	81/153

### Performance of the CNN compared with the orthopedic surgeon^s^

**Figure 2.**
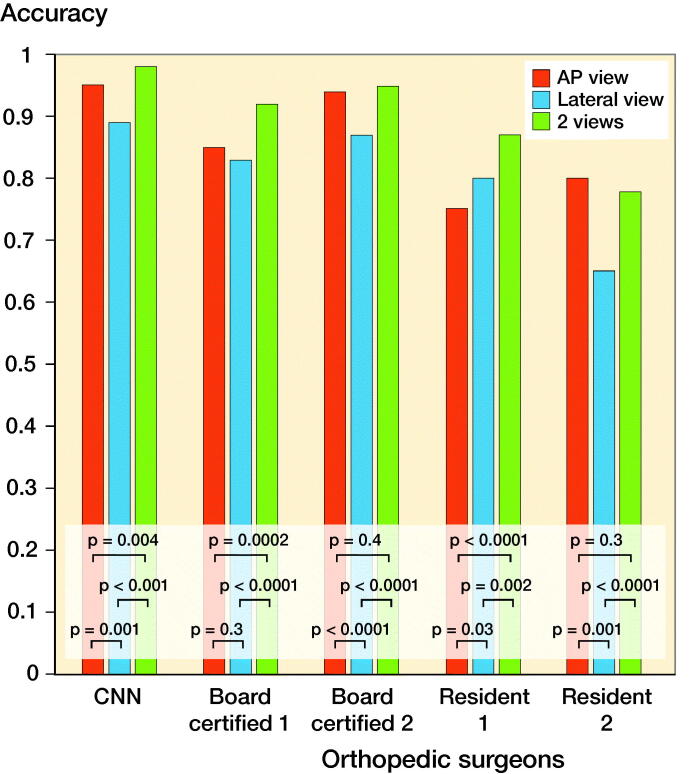
Comparison of the accuracy between the AP, lateral, and both views of the CNN and the 4 orthopedic surgeons. In the CNN model, the accuracy of the diagnosis based on both views was statistically better than the AP view alone and the lateral view alone. The accuracy of diagnosis based on the AP view alone was statistically better than the lateral view alone. The same trend was also seen with the board-certified orthopedic surgeons. AP = anteroposterior; CNN = convolutional neural network.

The accuracy of the CNN was comparable to or statistically significantly better than that of the orthopedic surgeons regardless of radiographic view used (Table 2 and Tables 3 and 4, see Supplementarys data). Improved recall and precision for the diagnosis of femoral neck fractures, trochanteric fractures, and non-fracture were found with the CNN model using 2 views compared with the AP or the lateral view alone (Table 5 and Tables 6 and 7, see Supplementary data). A comparison of the accuracy between the AP, lateral, and both views of the CNN and that of the orthopedic surgeons is shown in Figure 2. In the CNN model, the diagnostic accuracy based on both views was statistically better than that from the lateral view and the AP view. The interrater reliability of the orthopedic surgeons showed substantial to almost perfect agreement (Table 8).

**Table 2.** Accuracy, p-value of the accuracy compared with the CNN, average recall, precision, and F1 score of the diagnostic performance of the CNN and the 4 orthopedic surgeons based on both the anteroposterior and the lateral radiographs

CNN/ surgeon	Accuracy (CI)	p-value **^a^**	Average recall	Average precision	Average F1 score
CNN	0.98 (0.96–1.00)	–	0.98	0.98	0.98
Board certified					
1	0.92 (0.88–0.96)	0.01	0.92	0.92	0.92
2	0.95 (0.91–0.98)	0.1	0.95	0.95	0.95
Resident					
1	0.87 (0.82–0.93)	0.0006	0.87	0.89	0.88
2	0.78 (0.71–0.85)	< 0.0001	0.78	0.82	0.80

**^a^** compared with CNN

CI = 95% confidence interval;

CNN = convolutional neural network.

**Table 5.** Diagnostic performance of the CNN and the 4 orthopedic surgeons based on both the anteroposterior and the lateral radiographs

CNN/	Femoral neck fracture			Trochanteric fracture			Non-fracture		
surgeon	Recall	Precision	F1 score	Recall	Precision	F1 score	Recall	Precision	F1 score
CNN	1.00	0.98	0.99	0.94	1.00	0.97	1.00	0.96	0.98
Board certified									
1	0.96	0.89	0.92	0.94	0.92	0.93	0.86	0.96	0.91
2	0.96	0.96	0.96	0.96	0.96	0.96	0.92	0.92	0.92
Resident									
1	0.96	0.81	0.88	1.00	0.86	0.93	0.66	1.00	0.80
2	0.90	0.71	0.80	0.96	0.77	0.86	0.48	0.96	0.64

CNN = convolutional neural network.

**Table 8.** Interrater reliability presented with Cohen’s kappa of the orthopedic surgeons

	Board certified		Resident	
	orthopedic	surgeon	orthopedic	surgeon
Surgeon	1	2	1	2
Board certified				
1	–	0.85	0.78	0.73
2	0.85	–	0.76	0.78
Resident				
1	0.78	0.76	–	0.66
2	0.73	0.78	0.66	–

**Figure 3.**
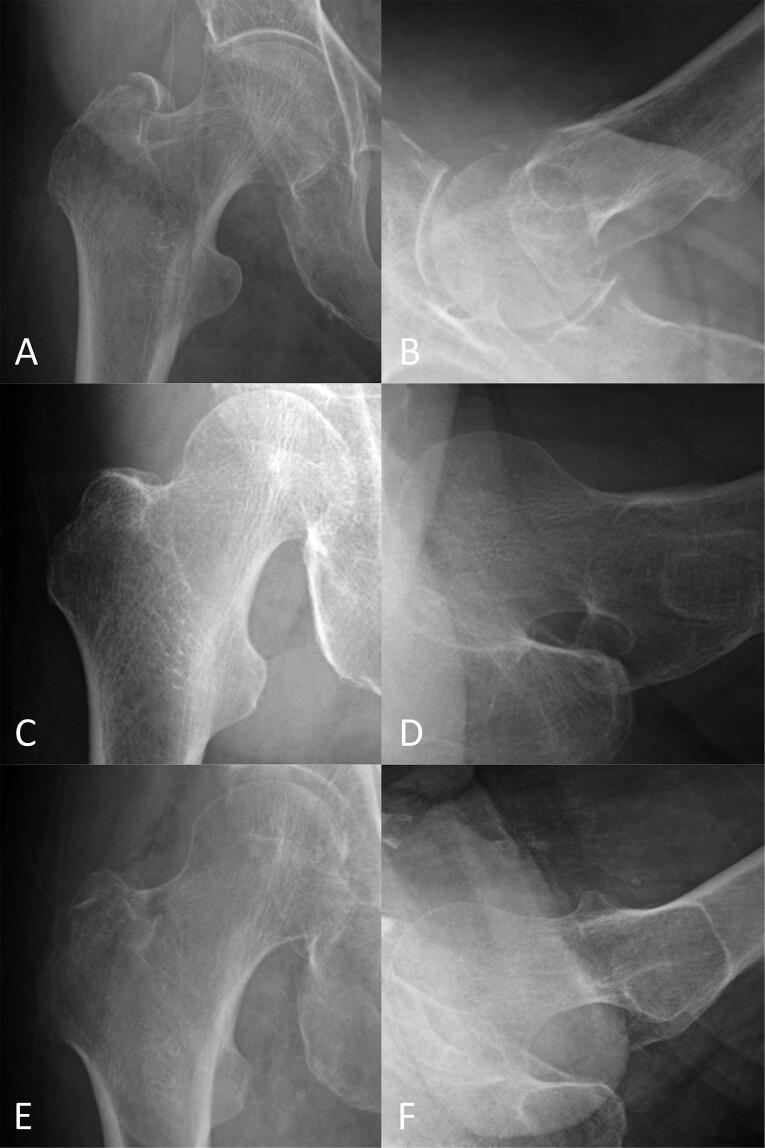
Representative radiographs of hip fractures. The AP (A) and lateral (B) radiographs of a trochanteric fracture, which the CNN misdiagnosed as a non-fracture, but all the orthopedic surgeon diagnosed correctly. The AP (C) and lateral (D) radiographs of a neck fracture, which 3 of the 4 orthopedic surgeons misdiagnosed as a non-fracture, but the CNN diagnosed correctly. The AP (E) and lateral (F) radiographs of a trochanteric fracture, which 3 of the 4 orthopedic surgeons misdiagnosed as a non-fracture or a neck fracture, but the CNN diagnosed correctly. AP = anteroposterior; CNN = convolutional neural network.

For reference, representative hip radiographs that were correctly diagnosed by the CNN but misdiagnosed by orthopedic surgeons and vice versa are presented in Figure 3.

## Discussion

We demonstrated that the ability of the CNN to differentiate femoral neck fractures from trochanteric fractures and non-fracture using AP and lateral hip radiographs was comparable or superior to that of orthopedic surgeons. The frontal radiograph was better for the extraction of the features for the CNN; however, adding the lateral radiographs to the decision process improved the recall and precision of the diagnosis.

The promising performance of our CNN to discriminate femoral neck fractures, trochanteric fractures, and non-fracture with an accuracy of 98% was demonstrated. The CNN model was trained on 1,553 AP hip radiographs and 1,070 lateral hip radiographs and validated on 150 AP and lateral hip radiographs. There are several previous studies using a CNN to diagnose hip fractures. Urakawa et al. (2019) presented a CNN model that predicted trochanteric fractures with an accuracy of 95.6% and an AUC of 0.984 using 2,678 images for training and 334 images for validation. Adams et al. (2019) reported a CNN model to diagnose femoral neck fractures with an accuracy of 94.4% and an AUC of 0.98 using 640 images for training and 160 images for validation. Chen et al. (2019) described a CNN model that predicted both femoral neck and trochanteric fractures while achieving an accuracy of 0.959 and an AUC of 0.98 based on 3,605 training images and 100 images for validation. Badgeley et al. (2019) used not only images, but also patient data and hospital process features as input for their CNN model, which achieved an accuracy of 85% and an AUC of 0.91. Although their CNN model’s accuracy and AUC were limited to 74% and 0.78, respectively, it was based solely on images, and was trained on 17,587 radiographs and 5,970 images for validation. The model presented in this previous study discriminated only femoral neck fractures versus non-fractures, trochanteric fractures versus non-fractures, or hip fractures versus normal hips, and did not distinguish femoral neck fractures from trochanteric fractures. It is clinically important, however, to diagnose hip fractures, including both femoral neck fractures and trochanteric fractures, and to correctly differentiate between the 2 conditions for appropriate surgical management.

The study highlights the importance of using both AP and lateral radiographs even with a CNN model to diagnose hip fractures. Acquiring 2 radiographs in 2 perpendicular directions facilitates the assessment of the relative positions for the 2 pieces of fractured bone (Plaats 1969). Occasionally, fractures are visible in only a single view. If that view is not obtained, then the examination will be interpreted as falsely negative. Radiology departments in most hospitals follow protocols that call for orthogonal views in frontal and lateral projections for the suspected fracture of long bones. Ensemble decision-making using the CNN model trained on both the AP and lateral radiographs improved the recall and precision of diagnosing femoral neck fractures and the trochanteric fractures. Using both the AP and lateral radiographs for training was also thought to contribute to the reduction in the number of images required to achieve the same or better accuracy than the previously reported model. However, there are few studies of deep learning in fracture detection using 2 or more radiographic views (Kitamura et al. 2019). It was easier to extract the fracture features from the AP radiograph for both the CNN and the orthopedic surgeons. This is why most of the previous studies achieved good accuracy using only AP radiographs for the training. The other reason to use only AP radiographs for training is because obtaining lateral radiographs of a non-fractured hip is difficult. In our study, we obtained lateral hip radiographs of non-fractured hips from patients who were suspected of having a hip fracture, but with a diagnosis of a sprain or bruise of the hip joint.

There are several limitations to our study. First, the number of images included was relatively small. To overcome this problem, we used both AP and lateral radiographs for training and also applied transfer learning and data-augmentation methods. Also, the validation dataset was relatively small. Further investigation in a larger cohort is warranted to better train and validate the CNN for improved diagnostic accuracy and reproducibility of hip fractures and type. Second, the CNN discriminated only among radiographs of femoral neck fractures, trochanteric fractures, and non-fracture. It could not diagnose pubic rami fractures, which also occur when elderly patients fall, and can cause pain near the hip joint. Third, the radiographs need to be cropped before inputting to the model. However, it is not difficult for even non-orthopedic surgeons to crop an image around the hip joint. Constructing an object detection model will solve the second and third limitations, but object detection is more difficult than image classification, as it must identify the accurate localization of the object of interest (Feng et al. 2019).

In conclusion, we have successfully demonstrated that the ability of the CNN to discriminate femoral neck fractures, trochanteric fractures, and non-fracture using both AP and lateral hip radiographs was comparable or superior to that of orthopedic surgeons. For the CNN, it was easier to extract the features of the fracture using the frontal radiograph; however, adding the lateral radiographs improved the recall and precision for diagnosing femoral neck fractures versus trochanteric fractures.

## Supplementary Material

Supplemental Material

## Supplementary data

Tables 3, 4, 6, and 7 are available as supplementary data in the online version of this article, http://dx.doi.org/10.1080/ 17453674.2020.1803664

Conception and design of this study: YY and SM. Acquisition of the datasets: YY, SK, HN, YI, TK, and TY. Training and validation of models: YY and SM. Analysis and interpretation of the results and data: YY, SM, SK, NY, JA, YS, and YK. Drafting the manuscript: YY and SM. Critically reviewing the manuscript: SK, YS, KI, SO, YE, and HT. Supervision: SM and SO.
