# Supplementary material for: Automated classification of hip fractures using deep convolutional neural networks with orthopedic surgeon-level accuracy: ensemble decision-making with antero-posterior and lateral radiographs
Source: Acta Orthop. 2020 Aug 12;91(6):699–704. doi: 10.1080/17453674.2020.1803664 (PMC8023868; doi:10.1080/17453674.2020.1803664)
Supplement: Supplemental Material [file IORT_A_1803664_SM7652.pdf]

Supplementary data

Table 3. Accuracy, p-value of the accuracy compared with the CNN, average recall, precision, and F1 score of the diagnostic performance of the CNN and the 4 orthopedic surgeons based on the anteroposterior radiographs alone

CNN/ surgeon	Accuracy (CI)	p-value [a]	Average recall	Average precision	Average F1 score
CNN	0.95 (0.91, 0.98)	–	0.95	0.95	0.95
Board certified					
1	0.85 (0.80 0.91)	0.004	0.85	0.86	0.86
2	0.94 (0.90–0.98)	0.7	0.94	0.94	0.94
Resident					
1	0.75 (0.68–0.82)	< 0.001	0.75	0.81	0.78
2	0.80 (0.74–0.86)	< 0.001	0.80	0.83	0.82

[a] compared with CNN
CI = 95% confidence interval;
CNN = convolutional neural network.

Table 4. Accuracy, p-value of the accuracy compared with the CNN, average recall, precision, and F1 score of the diagnostic performance of the CNN and the 4 orthopedic surgeons based on the lateral radiographs alone

CNN/ surgeon	Accuracy (CI)	p-value [a]	Average recall	Average precision	Average F1 score
CNN	0.89 (0.84–0.94)	–	0.89	0.89	0.89
Board certified					
1	0.83 (0.77–0.89)	0.2	0.83	0.83	0.83
2	0.87 (0.82–0.93)	0.7	0.87	0.88	0.88
Resident					
1	0.80 (0.74–0.86)	0.03	0.80	0.80	0.80
2	0.65 (0.57–0.72))	< 0.001	0.65	0.70	0.67

For abbreviations, see Table 2.

Table 6. Diagnostic performance of the CNN and the 4 orthopedic surgeons based on the anteroposterior radiographs alone

CNN/	Femoral neck fracture			Trochanteric fracture			Non-fracture		
surgeon	Recall	Precision	F1 score	Recall	Precision	F1 score	Recall	Precision	F1 score
CNN	0.94	0.96	0.95	0.92	1.00	0.96	0.98	0.89	0.93
Board certified									
1	0.92	0.84	0.88	0.98	0.83	0.90	0.66	0.92	0.77
2	0.96	0.92	0.94	0.94	0.98	0.96	0.92	0.92	0.92
Resident									
1	0.96	0.72	0.82	0.92	0.71	0.8	0.36	1.00	0.53
2	0.98	0.68	0.80	0.86	0.91	0.89	0.56	0.90	0.69

CNN = convolutional neural network.

Table 7. Diagnostic performance of the CNN and the 4 orthopedic surgeons based on the lateral radiographs alone

CNN/	Femoral neck fracture			Trochanteric fracture			Non-fracture		
surgeon	Recall	Precision	F1 score	Recall	Precision	F1 score	Recall	Precision	F1 score
CNN	0.88	0.86	0.87	0.90	0.90	0.90	0.88	0.90	0.89
Board certified									
1	0.80	0.82	0.81	0.92	0.82	0.87	0.78	0.87	0.82
2	0.82	0.91	0.86	0.88	0.92	0.90	0.92	0.81	0.86
Resident									
1	0.78	0.74	0.76	0.94	0.84	0.89	0.68	0.83	0.75
2	0.78	0.63	0.70	0.90	0.62	0.73	0.26	0.87	0.40

CNN = convolutional neural network.
